# Comparative gene co-expression network analysis of epithelial to mesenchymal transition reveals lung cancer progression stages

**DOI:** 10.1186/s12885-017-3832-1

**Published:** 2017-12-06

**Authors:** Daifeng Wang, John D. Haley, Patricia Thompson

**Affiliations:** 1 Department of Biomedical Informatics, Stony Brook University, Stony Brook, NY USA; 2 Stony Brook Cancer Center, Stony Brook Medicine, Stony Brook, NY USA; 3 Department of Pathology, Stony Brook Medicine, Stony Brook University, Stony Brook, NY USA

**Keywords:** Lung cancer, Epithelial to mesenchymal transition, Gene regulatory network, Comparative network analysis, EMT-dynamic signature genes, Cancer progression

## Abstract

**Background:**

The epithelial to mesenchymal transition (EMT) plays a key role in lung cancer progression and drug resistance. The dynamics and stability of gene expression patterns as cancer cells transition from E to M at a systems level and relevance to patient outcomes are unknown.

**Methods:**

Using comparative network and clustering analysis, we systematically analyzed time-series gene expression data from lung cancer cell lines H358 and A549 that were induced to undergo EMT. We also predicted the putative regulatory networks controlling EMT expression dynamics, especially for the EMT-dynamic genes and related these patterns to patient outcomes using data from TCGA. Example EMT hub regulatory genes were validated using RNAi.

**Results:**

We identified several novel genes distinct from the static states of E or M that exhibited temporal expression patterns or ‘periods’ during the EMT process that were shared in different lung cancer cell lines. For example, cell cycle and metabolic genes were found to be similarly down-regulated where immune-associated genes were up-regulated after middle EMT stages. The presence of EMT-dynamic gene expression patterns supports the presence of differential activation and repression timings at the transcriptional level for various pathways and functions during EMT that are not detected in pure E or M cells. Importantly, the cell line identified EMT-dynamic genes were found to be present in lung cancer patient tissues and associated with patient outcomes.

**Conclusions:**

Our study suggests that in vitro identified EMT-dynamic genes capture elements of gene EMT expression dynamics at the patient level. Measurement of EMT dynamic genes, as opposed to E or M only, is potentially useful in future efforts aimed at classifying patient’s responses to treatments based on the EMT dynamics in the tissue.

**Electronic supplementary material:**

The online version of this article (10.1186/s12885-017-3832-1) contains supplementary material, which is available to authorized users.

## Background

Lung cancer is the leading cause of cancer death in the United States with low 5-year survival of 17.7% despite earlier detection and advanced treatments [1]. Identifying novel mediators of tumor progression and treatment resistance for targeted intervention remains a major goal for treatment development.

The ability of tumor cells to undergo epithelial to mesenchymal transition (EMT) is a common feature of lung cancer cells that is associated with acquisition of ‘stem-like’ features [2, 3]. EMT, and its reversal MET, are complex dynamic processes whereby tumor cells undergo staged epigenetic reprogramming leading to acquisition of new traits and behaviors. While currently debated as to the necessity of E → M in metastatic tumor cell dissemination, undebated is that M-type lung cancer cells have stem-like features, exhibit enhanced drug resistance and demonstrate greater ability to migrate – all clinically significant biological changes that contribute to more aggressive/metastatic tumor behavior [4]. The complexity of the E → M shift is reflected in dramatic systematic changes in a dynamic fashion of developmental gene regulatory networks [5, 6].

Previous studies have detailed differentially expressed genes that distinguish the epithelial and mesenchymal states in non-small cell lung carcinoma (NSCLC) and serve to define signatures of EMT [5–9]. These EMT genes have also been reported to predict acquired drug resistance [10–15]. However, how the gene regulatory mechanisms act on gene expression transitions from E to M states, and the maintenance of these cell states, is still not well defined.

Recently, detailed temporal gene expression changes across multiple stages of EMT were measured, using RNA-seq in the mutant *KRAS* lung adenocarcinoma cancer cell lines H358 and A549 [6, 16, 17]. This provides a platform to analyze gene expression dynamic patterns specifically for lung cancer EMT. Here, we performed an integrated bioinformatics analysis for time-series gene expression datasets for H358 and A549 EMT with the intent to discover gene expression *temporal dynamic* patterns specific for EMT in lung cancer.

**Fig. 1:**
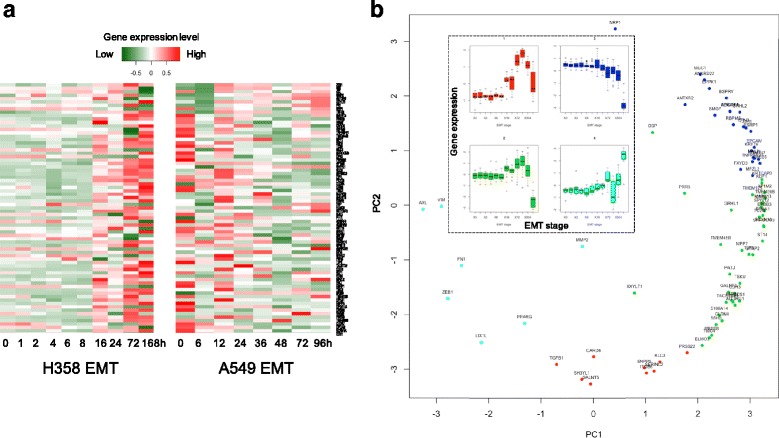
Previously identified EMT signature genes have distinct temporal expression dynamics during epithelial to mesenchymal transition in lung cancer. **a** The heatmaps show the normalized gene expression levels of 76 known EMT genes across H358’s ten EMT stages (left, 0 h, 1 h, 2 h, 4 h, 6 h, 8 h, 16 h, 24 h, 72 h, 168 h) and A549’s eight EMT stages (right, 0 h, 6 h, 12 h, 24 h, 36 h, 48 h, 72, 96 h) [16, 17]. These EMT genes were predicted according to their fold changes between epithelial and mesenchymal states only. Red: highly expressed. Green: lowly expressed. **b** PCA of 76 known EMT genes using their gene expression data in H358 EMT. The dots are genes. The x-axis is the PC1 coefficient, and the y-axis is the PC2 coefficient. The four gene groups have been clustered by K-means. The embedded boxplots display the gene expression level distributions across H358 EMT stages for four groups. The cyan group represents genes with an increasing expression pattern at middle EMT stages (~72 h and continuing) that includes the EMT associated EGFR resistance oncogene AXL [10]. The red group consists of EMT genes including TGFB1 having an increasing expression pattern at ~ 16 h which decays after 168 h. The gene expression in the green group increases slowly from 16 h but dramatically decreases after 168 h. The blue group includes genes that are decreasing in expression during EMT (from 24 h on)

We initially focused on a set of 76 genes previously reported to be the most differentially expressed EMT genes between E and M lung cancer states based on their expression fold changes, [10]. Focusing on these 76 EMT genes (Fig. 1), however, we discovered distinct EMT expression dynamic patterns when evaluated over a time series. Thus, to systematically reveal the gene expression dynamic patterns in EMT, we constructed gene co-expression networks, connecting genes if with high correlated expression profiles during EMT, and clustered the network into gene co-expression modules. Here we show that the modular eigengenes represent specific EMT expression temporal dynamic patterns on a transcript wide-scale. This enabled the identification of gene regulatory networks most consistent with networks involved in controlling the temporal EMT expression dynamic patterns; i.e., modular genes. Importantly these genes were highly correlated with the temporal patterns in both lung cancer cell lines suggesting that they represent a novel set of EMT-dynamic genes. Finally, we demonstrate the presence of temporal EMT-dynamic genes in lung cancer patient’s tumor tissues and show evidence of a relationship to patient outcomes not previously observed with the 76 EMT gene profile.

## Methods

### Time-series gene expression datasets during EMT in lung cancer

To systematically identify gene expression dynamic patterns common to NSCLC, we used time-series gene expression data from two lung cancer cell lines (H358 and A549) undergoing TGFbeta-induced EMT in this study. The data of H358 EMT includes a time-series of RNA-seq gene expression dataset derived from an inducible EMT model for which the H358 cells undergo TGFbeta1-induced EMT, including 12 time points (0, 1, 2, 4, 6, 8, 18, 24, 72, ~168, ~500 and >4300 h) during which EMT was monitored phenotypically [17]. The data of A549 EMT includes a time-series of RNA-seq gene expression dataset derived from an inducible EMT model for which the A549 cells undergo TGFbeta1-induced EMT, including 12 time points (0, 6, 12, 24, 36, 48, 72, 96 h) [16].

### Gene co-expression networks, modules and clustering for time-series gene expression data during EMT

We constructed the gene co-expression networks by connecting all possible gene pairs by edges with edge weights being the Pearson correlations of their time-series gene expression profiles during EMT for H358 and A549 EMT datasets. The gene co-expression networks were clustered by WGCNA (weighted correlation network analysis), R package [18] into the gene co-expression modules (minimum size = 100, scale-free fitting > 0.8). The eigengenes of modules, i.e., the first principal components of modular gene expression, were calculated and provided by WGCNA as well. An eigengene is a vector with its elements representing the expression levels at time points, and captures the most likely temporal gene expression changes across time points (dynamic pattern) of its co-expression module.

### Enriched pathways and functions of gene co-expression modules

To annotate the functions of gene co-expression modules, we calculated the enriched pathways and functions including KEGG pathways, REACTOME pathways and Gene Ontology (GO) terms for the genes of each gene co-expression modules using the web application, Database for Annotation, Visualization and Integrated Discovery (DAVID) [19], and R package, clusterProfiler [20].

### Identification of gene regulatory networks controlling gene co-expression modules

The gene regulatory network is represented as a two-column edge list in which the first column is the transcription factor (TF) and the second column in the target gene. We used the TF-target list as a reference gene regulatory network including all possible TFs and their target genes from the public datasets [21]. These TFs and targets were predicted using ChIP-seq experiments; i.e., the TFs have high binding signals at the promoter regions of their target genes. A TF is defined as the potential TF regulating a module if it has significantly numbers of modular target genes presenting in the reference regulatory network (hypergeometric test *p* < 0.05).

### Identification of hub genes in gene regulatory networks

The hub scores of genes in a gene regulatory networks are calculated as *hub_score*() function in R package, *igraph* [22]. The high score means that the gene has high influence over the network; i.e., the hub genes in the network.

### Gene knockdown experiments by RNAi

Lentiviral shRNAs (pRSI9-U6-(sh)-UbiC-TagRFP-Puro) were used to infect HCC4006 NSCLC cells in epithelial (E) and mesenchymal (M) cell states, with a minimum of five hairpins per gene. DNA bar codes were sequenced after nine cell doublings and normalized reads compared between the E and M states, expressed as log2 ratios.

### Gene expression normalization and identification of differentially expressed genes between TCGA lung cancer tissue samples and GTEx normal lung tissues

The gene expression data for 522 lung adenocarcinoma patient tissues in The Cancer Genome Atlas project (TCGA-LUAD) and 320 healthy lung tissues in Genotype-Tissue Expression (GTEx) project were normalized using R package, RUVseq [23]. The differential expression analysis that calculated the logFC values for all genes was completed using R package, edgeR [24].

### Definition of personalized EMT period (PEP) for TCGA lung cancer patients based on the expression of EMT-dynamic genes

Given a TCGA patient and an EMT time-series gene expression data (H358 or A549), we first calculated the Pearson correlation of his/her TCGA gene expression levels and each EMT stage’s gene expression levels of 254 EMT-dynamic genes (Additional file 1: Table S3) identified by our analysis, and then found the stage that has the maximum correlation, which was defined as the ‘EMT-stage’ for this patient; i.e., personalized EMT period (PEP).

### Principal component analysis (PCA) and survival analysis of TCGA lung cancer patients based on the TCGA expression of select genes

The PCA analysis was performed for 522 TCGA LUAD patients and the TCGA expression of select genes in TCGA using *prcomp* function in R. The select genes can be 254 EMT-dynamic genes, 76 previously known EMT genes, or all ~18,000 protein-coding genes. The first two principal components, PC1 and PC2 were selected, and the patients coefficients over PC1 and PC2 were the coordinates on PCA plots in Fig. 5a. The Kaplan-Meier survival analysis was performed, using R package, *survminer* for three major PEP clusters (patient numbers >10): 16 h, 72 h and 168 h. Their Hazard ratios were calculated regarding the PEP cluster 0–8 h.

## Results

### Known EMT signature genes have specific temporal expression dynamics during EMT

It is established that gene expression undergoes dynamic changes as tumor cells undergo EMT and that this is driven by alterations in the tumor gene regulatory mechanism in response to EMT promoting stimuli e.g., macrophage and platelet derived TGFbeta [25–27] or HGF [28]. To identify gene expression dynamic patterns common to NSCLC, we compared time-series gene expression data from two lung cancer cell lines (H358 and A549) undergoing TGFbeta-induced EMT (see Methods) [16, 17]. Using these lines, we identified common and represented temporally gene expression dynamic patterns from two datasets, revealing specific gene regulatory activities common between the different cell lines during EMT.

EMT involves an epigenetic based reprogramming of gene expression that occurs in over a 2 – 14 day time period depending on the inducer, cell model and culture conditions [29]. Previous work by comparing RNA abundance between NSCLC cell lines in distinct E and M states, led to the identification of 76 NSCLC EMT signatures genes [10]. Using our data, we found that the 76 EMT signature genes indeed show specific expression transition dynamics during EMT (Fig. 1). Further, using principal component analysis (PCA) and our time series, we identified four major groups of temporally-regulated genes among the EMT signature gene set: 1) a mid EMT stage (cyan) increasing at ~72 h and continuing over the time series that includes the EMT associated EGFR resistance oncogene AXL [10]; 2) genes increasing expression at ~ 16 h that decays after 168 h (red group); 3) a group of genes that increase slowly from 16 h but dramatically decrease after 168 h (green) and 4) a group of genes that includes genes that are declining in expression during EMT starting at 24 h (blue). These PCA-derived dynamic patterns of gene expression suggest that EMT state genes are differentially regulated in a coordinated fashion, forming co-regulated gene regulatory networks as cells progress from E to M.

### Gene co-expression network analysis reveals specific gene expression dynamic patterns of pathways and functions during EMT in lung cancer

Important to the discovery of gene effects on EMT, it is important to consider the gene regulatory networks controlling EMT signature genes, which act in turn to co-regulate other genes. Thus, we sought to identify all possible EMT expression dynamic patterns on a genome wide-scale to enhance discovery of novel gene regulatory network effects driving EMT in NSCLC at the system level. The presence of genes exhibiting similar dynamic expression profiles suggests that they are undergoing a coordinated regulation. i.e., the gene regulatory factors, including transcriptions factors, form a gene regulatory network that drive gene expression dynamics during EMT.

Studying individual gene expression is prone to noise making it more difficult to reliably identify expression dynamics and gene regulatory network effects at the system level. Thus, in this paper, we sought to study systematic gene expression dynamic patterns during EMT using a comparative gene co-expression network analysis across two mutant *KRAS* adenocarcinoma NSCLC cell lines undergoing TGFbeta-induced EMT, H358 and A549. The genes that have correlated expression profiles (i.e., co-expression) are those that are more likely co-regulated by similar regulatory mechanisms. This co-expression relationship was employed to identify functional groupings in EMT. Thus, we first identified the co-expressed genes during EMT by clustering gene co-expression networks into gene co-expression modules. Specifically, for each EMT cell line dataset (H358 or A549), we constructed the gene co-expression network in which genes are connected by edges with weights derived from the correlations of their expression profiles during EMT. Next the gene co-expression networks were clustered into gene co-expression modules using weighted correlation network analysis (WGCNA; described in the Methods section).

**Fig. 2:**
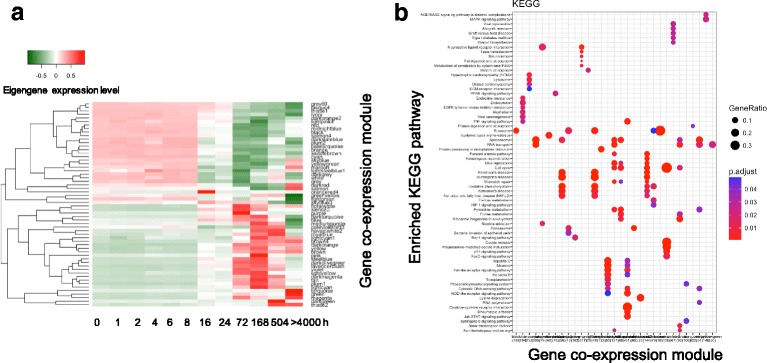
The eigengenes and enriched pathways of gene co-expression modules in H358 EMT. **a** The heatmap shows the eigengene expression levels across H358 EMT stages for 55 gene co-expression modules. Red: high expression level; Green: low expression level. These eigengenes represent the gene expression dynamic patterns at the system level in H358 EMT. The gene co-expression modules are identified using WGCNA [18]. **b** The enriched KEGG pathways of gene co-expression modules, which are found by clusterProfiler [20]. The rows are the enriched pathways, and the columns are modules. The dot size is proportional to the modular gene fraction involved in the pathway (i.e., number of pathway genes in the module over number of total pathway genes). The darkness of color is proportional to the enrichment score (adjusted *p* value)

Using this strategy, we identified 55 gene co-expression modules for the H358 cell line undergoing EMT (Additional file 1: Table S1). Figure 2a shows the eigengenes of these modules and illustrates the represented robust and systematic expression dynamic patterns of modular genes. We can see that the eigengenes display four major distinct expression dynamic patterns across different EMT stages (defined as time) and includes those that are: 1) down-regulated after 8 h; 2) up-regulated from 8 h to 16 h; 3) up-regulated from 72 h; and 4) up-regulated at very late stages, after 168 h. The presence of these different eigengene expression dynamic patterns suggests that specific underlying gene regulatory networks are being engaged during EMT; i.e., the expression changes at certain transition stages, especially 8-16 h.

To gain biological insight on EMT processes from these patterns, we analyzed the enriched pathways and functions among the genes of each module (Fig. 2b). We found for example that all the modules enriched with cell cycle related pathways and functions (i.e., “cell cycle modules”) were down-regulated after 8 h. This suggests that the cell cycle pathways tend to be down-regulated after 8 h during EMT in H358. We also found that the metabolic modules (i.e., the modules enriched with the genes involving in metabolic processes), in general, follow the eigengenes down-regulated at 8 h. Exceptions included the eigengenes of modules enriched with “glycerophospholipid metabolic process (*p* < 0.0001)”, “cholesterol metabolic process (*p* < 0.0001)” and “glyoxylate metabolic process (*p* < 0.0001)”, which were up-regulated at 72 h. These latter observations suggest that these specific metabolic pathways are potentially up-regulated during the late stages of EMT.

Because of recent interest in tumor immune regulating molecules and recent success with immunotherapies for some advanced lung cancer patients [30], we are interested in the expression dynamic patterns related to immune pathway related molecules during the EMT process. In our cell model system, we found that tumor cell immunological modules (i.e., enriched by immunological pathways) have eigengenes that are most commonly upregulated at late stages. The only exception was the “Leukocyte transendothelial migration module”. For example, modules enriched with “B cell receptor signaling pathway”, “Interferon signaling”, and “NOD-like receptor signaling pathway” were found to be up-regulated between 8 and 16 h, but the eigengene of the module enriched with “Platelet activation” is up-regulated at 72 h. These patterns suggest that immunological pathways are generally up-regulated during EMT and are most pronounced at late stages of EMT. The full list of enriched pathways and functions including KEGG, REACTOME and GO terms is included in the supplemental table.

Supporting our interpretation that the observed expression dynamics were EMT specific, we observed highly similar expression dynamic patterns from the analysis of eigengenes of the lung cancer cell line A549 EMT gene co-expression modules (54 modules in total, Additional file 1: Table S2) to that of H358. The cell cycle modules and most metabolic modules were similarly down-regulated from 12 h and immunological modules were up-regulated at late stages after 12 h (Supplemental Figures). Thus, using gene co-expression network analysis, we identified specific gene expression dynamic patterns during EMT on a genome scale and the transcriptional timing for enriched pathways and functions among co-expressed genes during experimentally induced EMT were shared across two different lung cancer cell lines.

### Identification of EMT-dynamic genes in lung cancer

**Fig. 3:**
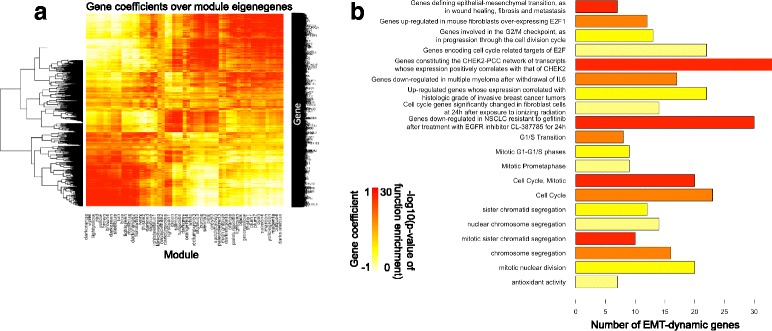
The gene coefficients over module eigenegenes, enriched functions and pathways of EMT-dynamic genes. **a** The heatmap shows the correlation matrix between ~18,000 genes and the eigengenes of 55 modules in H358 EMT. **b** The barplot shows the enriched pathways and functions (y-axis) of 254 EMT-dynamic genes. The x-axis is the number of EMT-dynamic genes for the enriched pathway/function. The colors correspond to the –log10(enrichment p value)

Here, we have observed that the genes that are highly correlated with modular eigengenes (e.g., large gene coefficients while projecting genes to eigengenes) follow strong and specific EMT gene expression dynamic patterns. Figure 3a displays the gene-eigengene correlation heatmap, where heatmap values represent the Pearson correlations coefficients between genes and modular eigengenes. For each module, there exists a number of genes having high modular memberships (large positive coefficients), which means that the enriched pathways and functions involve multiple genes. Also, each gene has high memberships in multiple modules; i.e., it has high correlations with multiple eigengenes. This suggests that any individual gene can participate in multiple pathways and functions during EMT. Notable, 36 of 55 H358 modules and 49 of 54 A549 modules have high correlations with at least one previously identified EMT signature genes (*r* > 0.9). We refer to these modules as *EMT modules*, suggesting that their eigengenes capture the specific EMT gene expression dynamic patterns. These results collectively demonstrate that EMT-specific dynamic expression patterns differ from the large fold-changes derived from transcriptomic studies of purer E and M type cells as two distinct states.

To genes with highest memberships, we selected the top 50 of genes with high memberships for each EMT module, and defined them as an extended set of novel EMT-dynamic genes. In total, we found that 254 genes display high memberships for both H358 and A549 EMT modules (Supplemental table). Thus, they show consistent EMT expression dynamics between the H358 and A549 undergoing EMT and are referred to as *novel EMT-dynamic signature genes* (Additional file 1: Table S3). Interestingly, they have no overlaps with the 76 previously identified EMT genes derived from large fold-change between E and M stages. This implies that the genes having large fold-changes between EMT endpoints may not be necessary to EMT-specific expression dynamics. Moreover, these EMT-dynamic genes also show gene expression transition dynamics during lung cancer EMT (Additional file 2: Figure S1). We further checked the enriched functions and pathways among the 254 EMT-dynamic genes. In Fig. 3b for example, EMT-defined genes, E2F targets and CHEK2 co-expressed genes are enriched. Moreover, many enriched functions and pathways are related to cell cycle, suggesting that the cell cycle genes exhibit strong expression dynamic patterns in lung cancer EMT. The relevance of this observation in terms of disease behavior however is not known.

### Computational prediction of gene regulatory networks driving the gene expression dynamics of pathways and functions during EMT

The various gene regulatory factors, including transcription factors, control the gene expression dynamics during EMT to coordinate biological phenotypes rather than acting randomly; i.e., they form a gene regulatory network. Thus, we sought to identify potential gene regulatory networks that drive specific EMT expression dynamics using computational approaches (Methods). The assumption here is that the genes that cluster together in a co-expression module are more likely to be co-regulated by similar gene regulatory mechanisms than those that are not cluster. As such, transcription factors that have significant numbers of their target genes in a given module are the most likely co-regulating gene involved in the control of the EMT expression dynamic patterns of that module.

To explore this assumption, we used a transcription factors-target list as a reference gene regulatory network. This included all possible transcription factors and their target genes from public datasets such as ENCODE [31]. These transcription factors and targets were predicted using ChIP-seq experiments; i.e., the transcription factors have high binding signals at the promoter regions of their target genes. Given a module, we define the potential transcription factors regulating the module based on significant numbers of their target genes in the module using transcription factors-target relationship from the reference gene regulatory network (hypergeometric test *p* < 0.05; see Methods). In particular, the transcription factors having either positively or negatively correlated gene expression profiles with the module eigengene during EMT are predicted as potential transcription factors regulating the modular genes along with the modular pathways and functions. Two specific examples are described: TF regulators of cell cycle gene changes in EMT and TF regulators of immune modules in EMT.

**Fig. 4:**
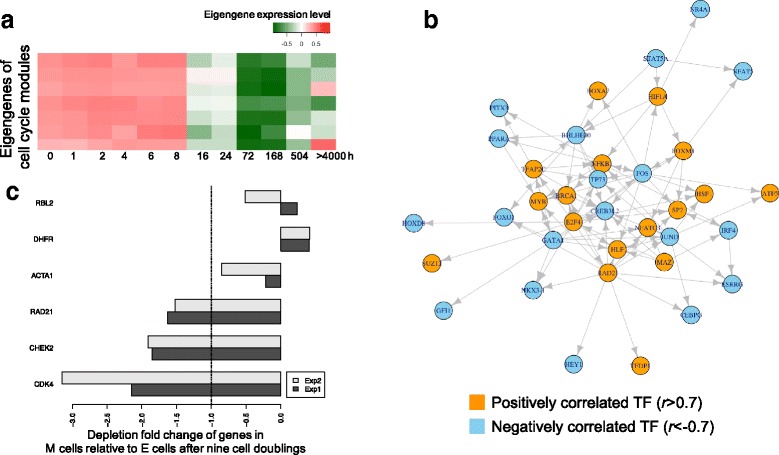
The gene expression dynamics and regulatory networks for cell cycle modules in H358 EMT. **a** The heatmap shows the eigengene expression level across H358 EMT stages for all 7 cell cycle modules. Red: high expression level; Blue: low expression level. **b** The predicted gene regulatory network controlling the cell cycle modules. Nodes are the transcription factors (TFs). The TFs in the network have significantly large numbers of target genes in the cell cycle modules (*p* < 0.05). The orange TFs have highly positive correlated expression with cell cycle eigengenes (Pearson correlation coefficient > 0.7), and the light-blue TFs have negatively correlation (Pearson correlation coefficient < −0.7). **c** The gene expression fold changes by RNAi depletion in M cells relative to E cells. The dashed line highlights the 1-fold of down-regulation

The EMT-dynamic genes show a strong relationship to cell cycle functions and pathways by gene set enrichment analysis (GSEA) (Fig. 3b). We found that essentially all cell cycle modules show a down-regulated expression pattern after 8 h (Fig. 4a). Using this information, we predicted a gene regulatory network consisting of TFs with significant numbers of targets in cell cycle modules, which also correlated with eigengenes of cell cycle modules (Fig. 4b). A number of the identified TFs appear to regulate each other; i.e., they work together to control the cell cycle in EMT in these cell lines. For example, we identified TF RAD21 as the top hub regulator in the network that was positively correlated with cell cycle expression dynamics in EMT (with highest hub score = 1, See Methods). In addition, the EMT-dynamic genes are enriched with CHEK2 co-expressed genes (Fig. 3b). While cell cycle shows a specific gene expression dynamic activity in EMT, interestingly EMT derived mesenchymal cells show sensitivity to pharmacological polo-like kinase inhibitors [32] and to shRNA inhibition of cell cycle checkpoints (Fig. 4c). We explored the impact of cell cycle modulation on E and M cells using two RNAi barcode experiments, each using 5 - 6 individual hairpins and associated barcodes per target. Following lentiviral infection with shRNA constructs targeting CHEK2, RAD21 and control targets (RBL2, DHFR, ACTA1), E and M cells were measured for DNA barcode depletion after nine cell doublings. When knockdown is deleterious to cell viability barcode sequences are depleted. Both experiments show that RAD21 and CHEK2 knockdown significantly depletes M cells relative to E cells (Fig. 4c), while knockdown of the control genes RBL2, DHFR and ACTA1 showed little effect.

Another example is the immunological module. As shown in Additional file 3: Figure S2, a gene co-expression module during H358 EMT is enriched with immunological pathway genes including interferon signaling, immune-regulatory interactions and cytokine signaling. The eigengene of this module represents the immunological EMT expression dynamic pattern, and shows an expression pattern upregulating after 8 h, especially from 72 h (Additional file 3: Figure S2A). Also, we found that several key transcription factors (TFs) involved in regulating immunological function genes including STAT3 (*r* = 0.93), ELK3 (*r* = 0.86) and IRF4 (*r* = 0.81) positively correlate with the module eigengene. In addition to these TFs, oncogenic and DNA damage transcription factors like JUND (*r* = 0.62), DDIT3 (*r* = 0.82) and FOS (*r* = 0.87) exhibited positive correlations with the eigengene of immunological module. However, the oncogene repressor, SIN3A was strongly inversely correlated (*r* = −0.81) with the module eigengene, suggesting that some oncogenes are upregulated at late stages of EMT due to the downregulation of SIN3A, which triggers the transcriptional activation of immunological pathways. In addition, TFs involved in regulating chromosomal structure such as SMC3 (*r* = -0.83) and POLR2A (*r* = -0.87) were also negatively correlated with the eigengene of immunological module. This suggests that the tumor immune modulating genes may potentially interact with chromosomal structures at late stages of EMT. Further and interesting, putative TFs co-regulating immunological gene expression dynamics during EMT form a structured gene regulatory network (Additional file 3: Figure S2B); i.e., the positively correlated TFs tend to regulate each other as well in a more connected sub-network (orange) than the negatively correlated ones.

### Revealing EMT periods of TCGA lung cancer patients using EMT-dynamic genes

Given the sharing of features between two cell lines, we next sought to assess if the EMT-dynamic genes identified using the time-series gene expression data of EMT-induced cell lines, H358 and A549, studied in culture were of any relevance to disease in patients. To assess this, we utilized gene expression data from The Cancer Genome Atlas (TCGA) project and Genotype-Tissue Expression (GTEx) projects, which provide publicly available genome-wide gene expression data for lung cancer tissues from 522 Lung Adenocarcinoma (LUAD) patients and normal tissues from 320 healthy individuals [33, 34]. First, we calculated the degrees of differential expression between TCGA and GTEx tissues for all genes; i.e., log (Fold Change) or log (FC) values (see Methods), and found that the EMT-dynamic genes have significantly larger log(FC) values than other genes (t-test *p* < 0.005). This analysis first established that the EMT-dynamic genes are significantly differentially expressed in lung cancer patient tissues. In addition, we also found that the EMT-dynamic genes correlate with the tumor purity. We used the tumor purity measurements for TCGA LUAD samples in [35], and correlated them with gene expression levels (FPKM values) across samples. It is interesting that the 254 EMT-dynamic genes have significantly higher correlations than other genes (t-test *p* < 7e-6 and ks-test *p* < 1e-8).

**Fig. 5:**
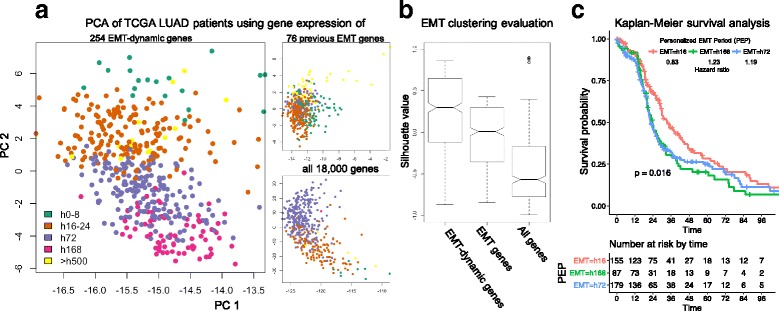
EMT-dynamic genes reveal EMT periods of TCGA lung cancer patients. **a** The PCA plots show the PC1 and PC2 coefficients of 522 TCGA LUAD patients using their TCGA gene expression data for three gene sets: 254 EMT-dynamic genes (left), 76 previously identified ETM genes (top right) and all ~18,000 genes (top bottom). The dots are patients. The colors denote the patients personalized EMT periods (PEPs) in H358 (Methods): green (h0-8), brown (h16-24), purple (h72), magenta (h168) and yellow (>h500). **b** To evaluate how well TCGA LUAD patients are clustered on Panel A using three gene sets, the boxplots show the silhouette value distributions. The 254 EMT-dynamic genes have significantly higher silhouette values than others. **c** The personalized EMT periods (PEPs) of 522 TCGA LUAD patients significantly classify the patient survival rates by the Kaplan–Meier analysis. Three major PEP groups are h16 (red), h168 (green) and h72 (blue). The reference group of hazard ratios consists of the patients whose PEPs is h0-8

Next, we were interested in whether EMT-dynamic gene expression in TCGA lung cancer patient tissue also exhibits evidence of EMT dynamics derived from cell line data. Specifically, we wanted to determine if the expression of EMT-dynamic genes identified in a time series study in vitro was present in lung cancer tissue samples across patients as evidence of similar temporal effects in lung tumor EMT evolution in the patient setting. Thus, for each TCGA patient, we correlated her/his TCGA gene expression data with each of the in vitro derived EMT transition period specific gene expression data for the 254 EMT-dynamic genes, and found the EMT transition period specific gene expression with maximum correlation (see Methods). We then classified each patient tissue sample to an EMT-dynamic gene defined as a ‘patients EMT transition period’ or PEP. As shown in Fig. 5a, the scatter plot shows the PCA coefficients of 522 TCGA patients with adenocarcinoma of the lung (LUAD) on the first two principal components of their TCGA gene expression levels of EMT-dynamic genes (FPKM values, See Methods). Both cell lines are adenocarcinoma type NSCLC. Patients with the same H358 PEP (same color) are clustered together. Moreover, and interestingly, PEP clusters corresponding to adjacent EMT periods in culture appear next to each other on PCA plot; i.e., at the patient level we observed that the PEP clusters, in general follow, temporal EMT progression patterns from 0 to 8 h, 16-24 h, 72 h, 168 h to >500 h that were identified in cell lines induced to undergo EMT. We did not observe this EMT progression pattern using the 76 previously identified EMT genes or all ~18,000 protein-coding genes (Fig. 5a). Also, the silhouette values of TCGA patients by 254 EMT-dynamic genes are significantly larger than the 76 previously identified EMT genes or all ~18,000 protein-coding genes. This suggests that the PEPs by our 254 EMT-dynamic genes cluster more significantly together and reveal the EMT developmental periods present in LUAD patients not observed using dichotomized E and M gene groups.

To explore clinical relevance, we assessed the relationship between PEPs and patient outcome. Survival plots (Fig. 5c) show that the survival rates of TCGA patients differ significantly when classified by PEPs (*p* < 0.016). The survival analysis includes three major PEP clusters for TCGA patients are present: h16, h72 and h168, and uses the PEP cluster h0-8 as reference to calculate the Hazard ratios. In addition to the significant classification, the order of three major PEP clusters also generally follows the EMT progression and lung cancer development; i.e., the patients in the PEP cluster h168, h72 and h16 have the lowest, middle and highest survival month distributions, respectively. However, when we use the previously identified 76 EMT signature genes or all ~18,000 protein coding genes to define the PEP for TCGA patients, this pattern is not present, and the patients are not significantly classified by the PEP either (*p* = 0.55 for 76 EMT signature genes; *p* = 0.2 for ~18,000 protein coding genes). Moreover, the identification of TCGA patients PEPs using our identified 254 EMT-dynamic genes is independent from the patient stage information provided by TCGA. Thus, this suggests that the in vitro identified EMT-dynamic genes capture aspects of the gene expression dynamics of EMT at the patient level, and the PEPs can be potentially used as additional feature at diagnosis to classify the patient outcome.

## Discussion

Gene signatures discriminating epithelial and mesenchymal cancer cell states have been very successful in categorizing cell lines and micro dissected or laser captured tumor tissues [36]. However, the ability to translate these signatures to clinical relevance as prognostic or predictive biomarkers or to guide target discovery for drug development has been less successful. Here we asked whether detailed time course data could be used to identify gene changes during EMT as a dynamic process and whether dynamic changes in gene expression during EMT in the in vitro setting can be extended to understand EMT states in vivo in patient populations.

To test this, we systematically analyzed the temporal gene expression dynamic patterns during the EMT in NSCLC using data derived from lung cancer cell lines induced to undergo EMT using data collected at multiple time points during the slow transition of cells going from E to M. Previously, we and others have shown that the EMT gene expression patterns are generally conserved between two EMT-induced lung adenocarcinoma KRAS mutant cell lines, H358 and A549 [6, 16, 17]. Here, we discovered an extended set of novel EMT signature genes that have high correlation to time-series expression profiles with temporal patterns during EMT in both cell lines. These have been designated as EMT-dynamic genes of NSCLC. Further, we show that these novel EMT-dynamic genes that appear to represent distinct stages in the process of EMT are differentially expressed in lung cancer patients using TCGA lung cancer patient tissue sample data. Further, classifying patients using a personalized EMT period (PEP) that represents the maximum correlated EMT stage, we found that the PEPs of patients generally follow an EMT temporal trajectory observed in cell culture. The evidence of distinct cell culture defined temporal stages of EMT in patients along with evidence that different patient groups match different temporal states with effects on outcome was somewhat surprising. This strongly supports the idea that gene expression dynamics patterns for EMT stages observed in cell culture are present in patients and that the relationship is not a simple E or M state. Most surprising was the finding of the temporally defined stages of EMT in the patient population using cross-sectional analysis suggesting that the temporal patterns observed in cell culture are occurring in human disease. While the clinical relevance of our findings remains to be determined, these data strongly suggest that patient groups differ significantly in relation to EMT status; a finding that if confirmed might explain heterogeneity in treatment sensitivity and guide drug development strategies aimed at capitalizing on knowing the tumor EMT stage at time of treatment. Promising is the observation that temporal studies that are easily conducted in cell culture may be of utility in modeling disease response and behavior in patients.

Here, we have constructed gene co-expression networks based on the gene-gene Pearson correlations during EMT, which captured the linear relationships. Given that the time points during EMT do not have equal intervals, the genes likely have non-linear co-expression relationships during EMT. Thus, the future work will integrate the non-linear and even causal relationships into our gene network analysis. In addition, the transformation of gene expression from the cell line to the tissue may not be linear, so in future, we can apply advanced machine learning methods such as tSNE [37] to better capture and translate the non-linear gene expression relationships from cell culture studies to patient tissue studies.

The gene expression dynamics during EMT is complex and controlled by gene regulatory networks. The gene regulatory networks consist of a variety of factors across multiple scales such as transcription factors, microRNAs, metabolites, etc. In this paper, we identified the gene regulatory networks based largely on transcription factor genes. Another important future work is to integrate multi-omics data as available including metabolomics and proteomics to systemically discover the gene regulatory networks and especially, find the druggable molecules and pharmacological approaches, based on their network positions to selectively target the epithelial to mesenchymal transition stages in lung cancer patients. With increasing large-scale multi-omics cancer data, our computational analysis in this paper can be used as a general-purpose tool to reveal the multi-omics biomarkers of epithelial to mesenchymal transition for additional cancer types.

## Conclusion

The specific gene regulatory networks control the epithelial to mesenchymal transition (EMT), a key process driving the lung cancer progression and drug resistance. We systematically identified a set of novel EMT-dynamic signature genes with specific expression dynamic patterns during the EMT process, using comparative gene co-expression network analysis for in-vitro time-series gene expression data of lung cancer cell lines H358 and A549 induced to undergo EMT. These genes dynamic patterns support the presence of differential activation and repression timings at the transcriptional level for various pathways and functions during EMT. We also validated the EMT activities of the hub regulatory genes of EMT-dynamic genes using RNAi. Moreover, we translated EMT-dynamic genes to TCGA patient data to reveal their clinical relevance, and found their ‘developmental period’ information for EMT that is present in lung cancer tissue derived from patients and positive association with patient outcomes supporting the potential in vivo significance of EMT-dynamic genes. Our work suggests that they capture the gene expression dynamics of EMT at the patient level, and can be used as additional prognostic or predictive biomarkers at diagnosis to classify the lung cancer patient outcome.

## Additional files

Additional file 1: Table S1. Genes and gene co-expression modules by WGCNA in H358 EMT. **Table S2.** Genes and gene co-expression modules by WGCNA in A549 EMT. **Table S3.** 254 EMT-dynamic genes (XLSX 612 kb)

Additional file 2: Figure S1. Novel EMT-dynamic genes have distinct temporal expression dynamics during epithelial to mesenchymal transition in lung cancer. PCA of 254 EMT-dynamic genes using their gene expression data in H358 EMT. The dots are genes. The x-axis is the PC1 coefficient, and the y-axis is the PC2 coefficient. The four gene groups have been clustered by K-means. The embedded boxplots display the gene expression level distributions across H358 EMT stages for four groups. The blue group represents genes with an increasing expression pattern at middle EMT stages (~72 h and continuing). The green group has an increasing expression pattern at ~ 16 h which decays after 168 h. The gene expression in the cyan group increases slowly from 16 h and varies until dramatically decreasing after 168 h. The red group includes the genes that are decreasing in expression during EMT (from 8 h on). (PDF 233 kb)

Additional file 3: Figure S2. The gene expression dynamics and regulatory networks for the immunological module in H358 EMT. (A) The heatmap shows the module’s eigengene expression level across H358 EMT stages. Red: high expression level; Blue: low expression level. (B) The predicted gene regulatory network controlling the cell cycle modules. Nodes are the transcription factors (TFs). The TFs in the network have significantly large numbers of target genes in the immunological module (*p* < 0.05). The orange TFs have highly positive correlated expression with the immunological eigengene (Pearson correlation coefficient > 0.7), and the light-blue TFs have negatively correlation (Pearson correlation coefficient < −0.7). (PDF 244 kb)

## Abbreviations

EMT: Epithelial to mesenchymal transition
ENCODE: Encyclopedia of DNA Elements
GTEx: Genotype-tissue expression
LUAD: Lung adenocarcinoma
NSCLC: Non-small cell lung carcinoma
PEP: Personalized EMT period
TCGA: The Cancer Genome Atlas
WGCNA: Weighted correlation network analysis
