# Supplementary figures and images for: Comparative gene co-expression network analysis of epithelial to mesenchymal transition reveals lung cancer progression stages
Source: BMC Cancer. 2017 Dec 6;17:830. doi: 10.1186/s12885-017-3832-1 (PMC5719936; doi:10.1186/s12885-017-3832-1)

# 254 EMT-dynamic genes

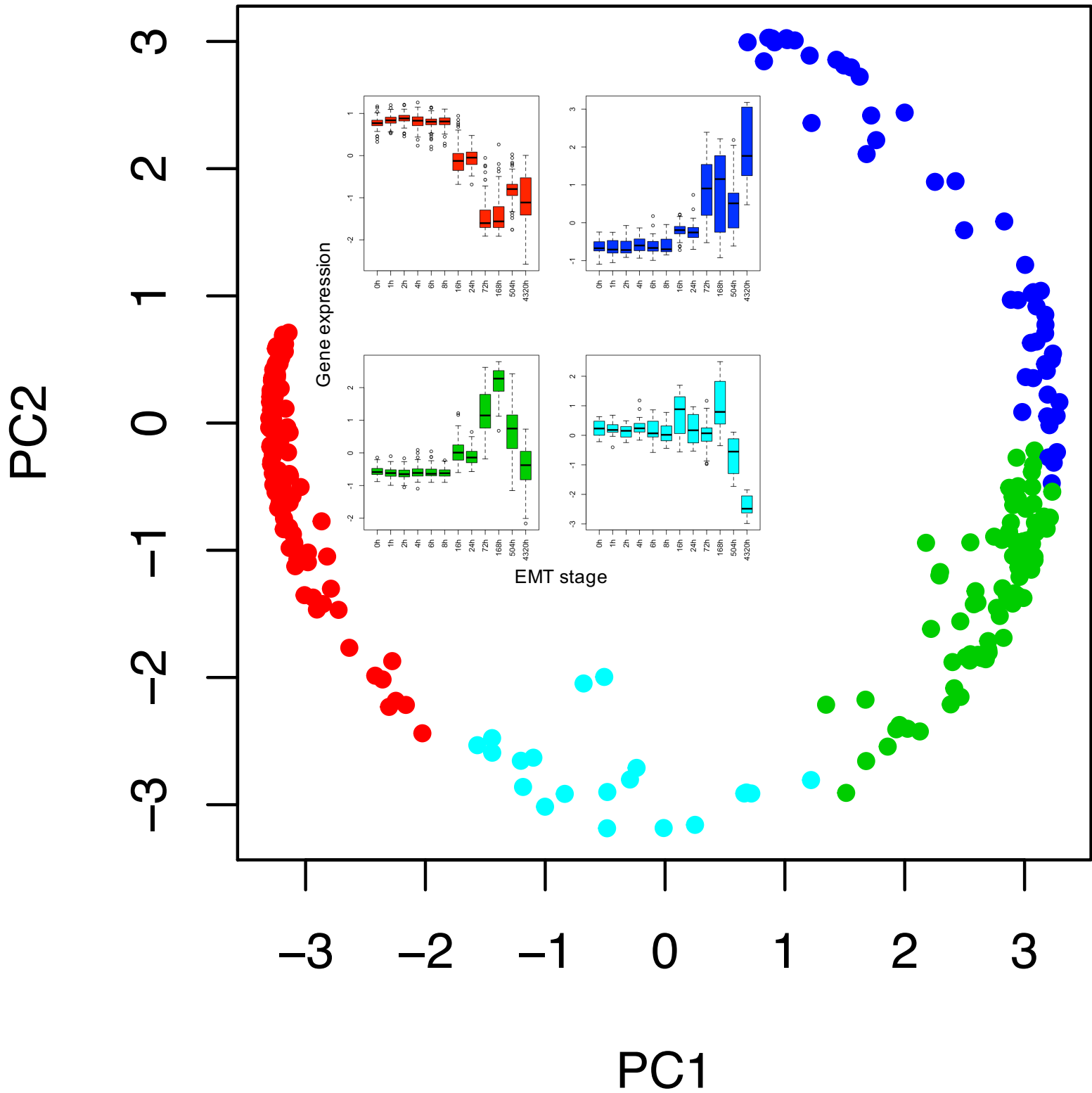

Supplement: Supplementary file 2 — Novel EMT-dynamic genes have distinct temporal expression dynamics during epithelial to mesenchymal transition in lung cancer. PCA of 254 EMT-dynamic genes using their gene expression data in H358 EMT. The dots are genes. The x-axis is the PC1 coefficient, and the y-axis is the PC2 coefficient. The four gene groups have been clustered by K-means. The embedded boxplots display the gene expression level distributions across H358 EMT stages for four groups. The blue group represents genes with an increasing expression pattern at middle EMT stages (~72 h and continuing). The green group has an increasing expression pattern at ~ 16 h which decays after 168 h. The gene expression in the cyan group increases slowly from 16 h and varies until dramatically decreasing after 168 h. The red group includes the genes that are decreasing in expression during EMT (from 8 h on). (PDF 233 kb) [file 12885_2017_3832_MOESM2_ESM.pdf]
