# Supplementary material for: Comparative gene co-expression network analysis of epithelial to mesenchymal transition reveals lung cancer progression stages
Source: BMC Cancer. 2017 Dec 6;17:830. doi: 10.1186/s12885-017-3832-1 (PMC5719936; doi:10.1186/s12885-017-3832-1)
Supplement: Supplementary file 3 — The gene expression dynamics and regulatory networks for the immunological module in H358 EMT. (A) The heatmap shows the module’s eigengene expression level across H358 EMT stages. Red: high expression level; Blue: low expression level. (B) The predicted gene regulatory network controlling the cell cycle modules. Nodes are the transcription factors (TFs). The TFs in the network have significantly large numbers of target genes in the immunological module (p < 0.05). The orange TFs have highly positive correlated expression with the immunological eigengene (Pearson correlation coefficient > 0.7), and the light-blue TFs have negatively correlation (Pearson correlation coefficient < −0.7). (PDF 244 kb) [file 12885_2017_3832_MOESM3_ESM.pdf]

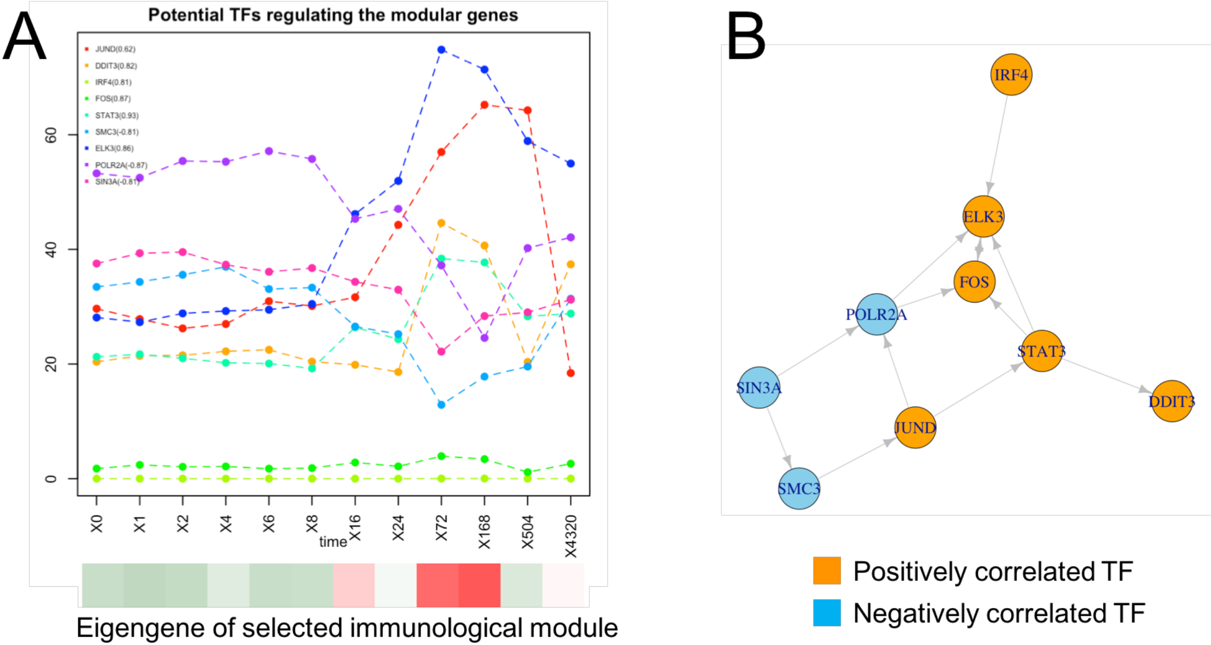
A
Potential TFs regulating the modular genes
JUND(0.62)
DDIT3(0.82)
IRF4(0.81)
FOS(0.87)
STAT3(0.93)
SMC3(-0.81)
ELK3(0.86)
POLR2A(-0.87)
SIN3A(-0.81)
0
20
40
60
X0
X1
X2
X4
X6
X8
X16
X24
X72
X168
X504
X4320
time
Eigengene of selected immunological module
B
IRF4
ELK3
FOS
POLR2A
STAT3
SIN3A
JUND
DDIT3
SMC3
Positively correlated TF
Negatively correlated TF
